# Exploring media representation of the exotic pet trade, with a focus on welfare: taxonomic, framing and language biases in peer-reviewed publications and newspaper articles

**DOI:** 10.1098/rsos.240952

**Published:** 2025-03-05

**Authors:** Jon Bielby, Gail Austen, Kirsten McMillan, Shannen Wafflart

**Affiliations:** ^1^ School of Biological and Environmental Sciences, Liverpool John Moores University, Liverpool, UK; ^2^ DICE, University of Kent, Canterbury, UK; ^3^ Dogs Trust, London, UK

**Keywords:** conservation, exotic pet trade, framing, reptiles, welfare, wildlife trade

## Abstract

The trade in non-domesticated animals for pets (exotics) is a global industry with considerable implications for a range of taxa and stakeholders. The scale of the trade means it receives coverage in both popular and scientific media, and some narratives may receive more attention than others. As these media play an important role in shaping public opinion, policy and legislation, we should consider and acknowledge biases and language use when reporting on the exotic pet trade. We use 320 peer-reviewed journal articles, and 191 newspaper articles on the trade between 2001 and 2020 to investigate how the trade is framed, focused and communicated within and between media types, paying particular attention to animal welfare as a framing. Our results suggest consistent variation in reporting of the trade within and between media types, with aspects of welfare being under-represented in peer-reviewed articles, while it was the most common focus of newspaper articles. If the exotic pet trade is to develop into a more ethical, sustainable sector, then reassessing our narratives, addressing knowledge gaps and identifying how communication styles can lead to desired behaviour change will be essential parts of the process.

## 1. Background

The global pet trade in non-domesticated companion animals is estimated to be worth billions of US dollars per annum, comprising millions of individuals across a broad range of taxonomic groups [1]. In this article, we follow notable previous studies and refer to animals without a long history of domestication that are kept as pets as ‘exotic’ [2–4]. Due to its size and diversity, the impacts of the trade are felt at a range of scales, from the individual animal to the ecosystem [5] and can come with complex costs and benefits. The trade can be economically and culturally important [6,7] and can provide sustainable livelihoods to communities within some regions [8,9]. It can also enrich the lives of pet owners [7,10,11], and advances in techniques and knowledge sharing have been beneficial to conservation efforts supporting *in situ* populations [12]. On the flip side, detrimental effects of the pet trade include population decline of source animals [13–16], spread of infectious disease [17], invasive species causing significant damage to native species and ecosystems [3], and traded animals suffering reduced welfare [12].

The complex costs and benefits associated with the trade provide the opportunity for information and content to be communicated from various angles. In this article, we refer to these as different framings (e.g. conservation or welfare), which refers to the way in which certain aspects of the subject may be highlighted to be more memorable or salient to the reader [18]. Interpreting, framing and communicating the trade in exotics can be subjective but incredibly important [19–21], as all types of media help form our perceived reality [18], shaping public perception and attitudes surrounding the urgency and magnitude of such issues [22,23]. The use of different framings may be highly dependent upon the interests and beliefs of the author(s) [20], the target audience and the objective of the research [24]. Similarly, the language we use affects our perception of society and can be used to alter behaviour [25], feeding into our actions as consumers, traders, researchers, journalists, voters and policymakers [26]. Consequently, we need to consider and understand framing and language use, conscious or otherwise, within communications on the exotic pet trade and how this varies across taxonomic groups and different media types.

Newspaper articles often including a ‘story’ element, which is designed to elicit an emotional response [27], commonly with reference to an individual (whether it be a human or an animal) [28]. As such, newspaper articles are likely to use different framings than peer-reviewed papers both because of this story element and depending upon what is more likely to catch the interest of the more general readership [27]. This contrasts with the peer-reviewed scientific literature, which tends to purposely avoid this style of writing [29]. Over a decade ago, a systematic review on welfare within wildlife trade highlighted several important knowledge gaps and biases within the peer-reviewed and grey literature [30]. The review identified that most articles were centred around the conservation of the traded species (71%), with only 17% of papers containing references to animal welfare. In contrast, recent research into newspaper coverage of illegal trade in US turtles outlined how welfare was a very common framing in US news media [21]. We may, therefore, expect newspaper articles to show different patterns in their framing compared with peer-reviewed papers based on a similar subject matter.

Increasingly, as the pet trade and related policies come under scrutiny in several countries [31,32], peer-reviewed papers, grey literature and popular media play a key role in shaping legislation via both public opinion and direct reporting to governmental bodies. In this article, we therefore aim to provide an overview of how the exotic pet trade is communicated in both the peer-reviewed literature and in newspaper articles with a specific focus on the use of welfare as a framing tool and its prevalence in both types of literature. First, we determine which framings are most widely used and compare their frequency both within and between media types, with a focus on animal welfare. Second, we used natural language processing techniques to summarize how language use varied between the two media types, specific to the different framings. Additionally, for peer-reviewed literature we also highlight taxonomic biases and describe how they covary with the framing category used and investigate whether those two factors cause variation in the engagement with a peer-reviewed paper. Drawing on previous research on the communication of the wildlife trade [30], we expected that peer-reviewed articles would be more frequently centred around conservation while welfare would be under-represented. We also predict that they would more commonly focus on taxa that are generally considered more relatable and charismatic (e.g. mammals and birds [33]). In contrast, based on analyses of illegal wildlife trade in newspaper articles [21], we expected that welfare would feature more commonly as a frame in this media, which would also use more emotive language than peer-reviewed papers [27]. By analysing and evaluating these two media types in this way, this article seeks to provide an overview of the biases and knowledge gaps in the reporting of the exotic pet trade. The gaps identified may have important implications for attitudes within society, the level of support for interventions and the evidence base underpinning any such changes. Further, it can provide us with a baseline for discussions on the effectiveness of current communication styles, and how they may be optimized to foster sustainable behaviour in specific stakeholder groups.

## 2. Methods

### 2.1. Collating articles and defining frames and taxonomic focus: peer-reviewed literature

We developed search terms in Scopus and Web of Science databases limiting the search to years from 2001 to 2021 inclusive (details in electronic supplementary material, S1). We focused on articles published in English because during this period it was the most consistently used language in scientific publishing, which came with associated problems [34]. Full details of the search terms and processes followed are outlined in electronic supplementary material, tables S1 and S2 and figure S1. Throughout the data collection process, PRISMA (preferred reporting items for systematic review and meta-analyses) [35] was used.

We defined peer-reviewed publications and their framing largely following Baker *et al.*’s analysis of literature on the wildlife trade [30] and via preliminary investigation of the publications yielded by the literature search. This resulted in five non-mutually exclusive framing categories: ‘Conservation’, ‘Economics’, ‘Welfare’, ‘Disease’ and ‘Invasive Species’. The first four were taken from Baker’s analyses, and the latter category was inductively identified due to its frequency in the literature on the pet trade and the large ecological cost it incurs [3]. To allocate peer-reviewed publications into framing categories we (J.B. and S.W.) iteratively developed a list of search terms (electronic supplementary material, table S3), the presence of which in the title, abstract and keywords was used to allocate articles to categories. We focused on these sections rather than the whole text because they are the most immediate way in which a reader will encounter a peer-reviewed paper and the way it is framed. If a publication fit into more than one framing category the paper was allocated to a separate ‘Multiple’ grouping, and if no criteria were met it was allocated to ‘No/other frame’. For peer-reviewed literature individual keyword searches were used to identify the taxonomic focus (electronic supplementary material, table S4). A ‘Multiple’ grouping was used for articles with multiple taxonomic foci, and an ‘Other’ category if none of the search terms were met.

## 3. Collating articles and defining frames: newspaper articles

We chose newspapers articles (print and electronic) as a representative of popular media for our investigation because they have been widely available and read during the study period compared with other possible routes (e.g. social media, online forums). The search terms ti(‘exotic pet*’) AND ti(‘trade’) were used to interrogate the ProQuest database for entries of English language newspaper articles over a comparable time period (1 January 2001–31 May 2021 inclusive). Full details of the methodology are outlined in electronic supplementary material, S1. A list of the details (e.g. newspaper title, article title, date of publication, article text) of resulting newspaper articles will be available in the electronic supplementary material. The aim of these search terms was to ensure that we captured newspaper articles covering the exotic pet trade specifically, rather than other types of trade in wildlife and its products. However, we acknowledge that in our effort to exclude non-pet-trade-related papers we may also have filtered out other relevant papers.

To provide a comparison with peer-reviewed publications, we examined newspaper articles, applying the same five framing categories to the entire body of text. Additionally, via inductive processing we identified five extra categories that differed from those in peer-reviewed papers: ‘Laws and Regulations’, ‘Public Health and Safety’, ‘Irresponsible Pet Ownership’, ‘Illegal Trade’ and ‘Defence of Trade’. In developing these categorizations, we feel that we have captured the main themes related to the trade, and that they were an effective way to categorize the articles collated given the existing biases in the literature.

In contrast to peer-reviewed abstracts, when ‘coding’ frames used within newspaper we identified sections of text, rather than single, relevant words in isolation because preliminary investigation suggested that, due to the more context-dependent writing style of newspapers, sections of text would be a more effective way of categorization. Frequency of newspaper articles within a framing category (‘Files’) and frequency of mentions within the article (‘References’) were calculated for each frame, and the content of each article was extracted for further analysis regarding language use and narrative. Full details of the process followed for thematic analysis can be found in electronic supplementary material, S1.

## 4. Comparisons and statistical analyses

### 4.1. Frequency of frames and taxonomic foci in peer-reviewed literature

G-tests were used to analyse variation in the number of peer-reviewed publications in each taxon–framing combination. Distributions of the count of peer-reviewed publications per year were non-normal. Consequently, to analyse whether they varied with each of framing category and taxonomic focus separately we used non-parametric Kruskal–Wallis tests followed by Dunn’s multiple comparison *post hoc* tests. All statistical analyses were conducted in the software package R [36]. Because of the thematic approach to analysis of newspaper article framing we decided to follow a more qualitative, non-statistical approach to this media type.

### 4.2. Factors associated with citations per year

We calculated the number of citations per year to be used as a response variable in a mixed-effects model using the R package lme4 [37]. Year of publication was included as a random effect, and framing category and taxonomic focus were included as fixed effects. We also included as fixed effects the impact factor of the journal and the h-index of the lead author (as listed on Scopus), as they are likely to play an important role in the citation rate. Mean citations per year and journal impact factor were log-transformed to make the distribution of the residuals meet the assumptions of the model. Model outputs were compared using Akaike information criterion (AIC) and models within 6 units of each other were considered to be equally supported [38]. Individual variable importance was judged based on the F-values from the overall ANOVA table obtained using the lmerTest library [39], and associated parameter estimate effect sizes and *t*-values in the model. The analysis of engagement was not conducted for newspaper articles as there was no obvious comparable metric to use to accurately quantify this.

## 5. Comparison of language between the two media types

To examine how language use varied among framings, taxa and media types we used two general-purpose lexicons: AFINN [40] and NRC [41] via the tidytext package [42]. Both lexicons contain many English words and are based on single words, which are assigned scores for positive/negative sentiment. The AFINN lexicon assigns words with a score of −5 to 5, with negative scores indicating negative sentiment and positive scores indicating positive sentiment. The NRC lexicon provides a finer level of detail and categorizes words into the following: positive, negative, anger, anticipation, disgust, fear, joy, sadness, surprise and trust. Both lexicons were implemented in this project to maximize opportunity to explore variation in measures of language use and sentiment between framings and media types. As metrics for comparison of categories, we calculated the mean net AFINN scores across all articles, while we calculated the mean percentage of words within each NRC category. For newspaper articles, the whole body of text was analysed in this way, whereas for the peer-reviewed publications only the abstract was used. Because of the qualitative approach we used in investigating newspapers generally and the differing sizes of the bodies of text analysed, we preferred not to run formal statistical analyses comparing language use between media types, and instead compared the range of values. Packages tidytext [42] and tm [43] were used for text mining. Figures were produced using ggplot2 [44].

## 6. Results

Our search resulted in 320 suitable peer-reviewed publications focused on the exotic pet trade between 2001 and 2021 (the dataset is included in electronic supplementary material, S2). Data from 2021 were excluded from any analyses relying on a full year of data (e.g. papers per year and citations per year), resulting in *n* = 305 for those. Peer-reviewed publications per year showed a large increase in number ranging from single figures in 2011, to a high of 53 in 2020, although this absolute change does not account for the increasing number of publications in general over this time (electronic supplementary material, figure S2).

### 6.1. Frequency of frames and taxonomic foci in peer-reviewed literature

G-tests suggest that counts of peer-reviewed publications were not evenly distributed across combinations of framing and taxon (table 1), both including data on a single category/focus (G = 36.89, *Χ*^2^ d.f. = 16, *p* = 0.002) and those within multiple categories/foci (G = 78.97, *Χ*^2^ d.f. = 36, *p* < 0.001). The largest general patterns to be noted are: (i) most peer-reviewed publications were framed in multiple ways, featuring multiple taxonomic groups, and (ii) relatively few were framed within a singular category focused on ‘Welfare’ or ‘Economics’.

The number of peer-reviewed publications were not distributed evenly across framings. Across the entire study period the following frequency of framings was observed: 55.3% ‘Multiple frames’ (*n* = 177), 19.1% ‘Conservation’ (*n* = 61), 7.8% ‘Disease’ (*n* = 25), 7.5% ‘Invasive species’ (*n* = 24), 6.6%, ‘No framing’ (*n* = 21), 3.1% ‘Welfare’ (*n* = 10), 0.6% ‘Economics’ (*n* = 2). Within the 177 ‘Multiple frames’ peer-reviewed publications 11 combinations were present: with the most frequent combinations including Conservation-Invasion (*n* = 29), Conservation-Economy (*n* = 21) and Disease-Welfare (*n* = 10). In addition, 45.2% (*n* = 80) of the 177 ‘Multiple frame’ papers included three or more framings. A full list of all combinations of framings and their frequencies are available in electronic supplementary material, table S5. Temporally, the number of papers based around Multiple framings, Conservation and Disease appeared to show the largest increases over the period covered by the study (electronic supplementary material, figure S3).

**Table 1.** Raw count of peer-reviewed publications in each combination of the framing categories and taxonomic foci.

	amphibian	aquatics	birds	mammals	multi taxa	other	reptile	total
Conservation	1	2	19	3	21	6	9	61
Disease	2	5	0	4	4	1	9	25
Economics	0	0	1	0	0	1	0	2
Invasive	0	7	2	2	3	6	4	24
Multiple	12	21	38	14	46	25	21	177
No/other frame	0	0	4	3	6	8	0	21
Welfare	0	1	1	1	2	4	1	10
total	15	36	65	27	82	51	44	

**Figure 1.**
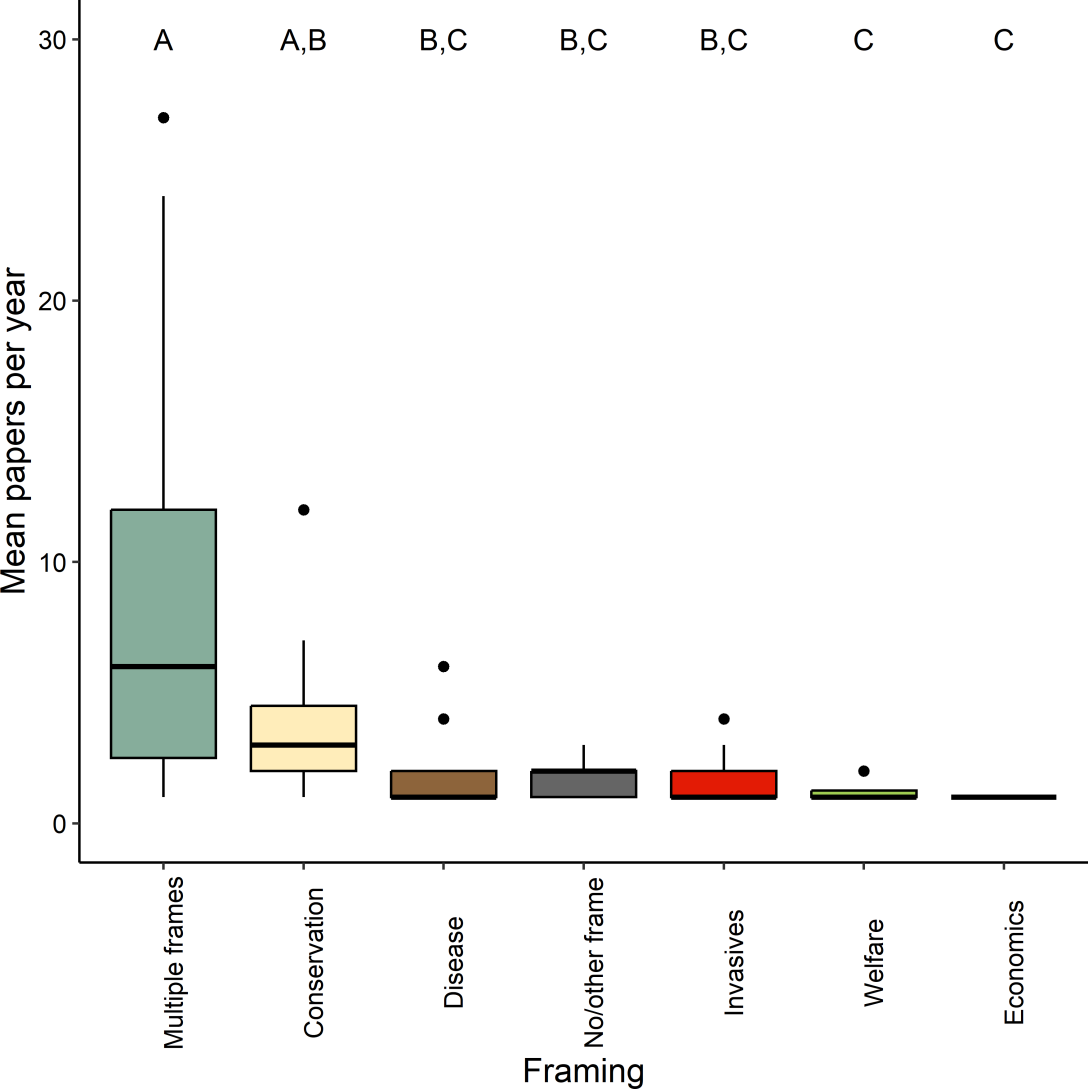
Mean peer-reviewed papers, per year, including the framing categories: Conservation, Disease, Economics, Invasives, Welfare, Multiple frames or No/other frame. Upper case letters indicate the grouping of framings based on significant differences found in a Kruskal–Wallis test.

Kruskal–Wallis analysis of the number of peer-reviewed publications per year highlighted significant differences between framings (*Χ*^2^ = 47.22, d.f. = 6, *p* < 0.001; figure 1). *Post hoc* Dunn’s tests suggest that the most common framing was ‘Multiple frames’ (median = 6.00, interquartile range (IQR) = 9.50), which was used significantly more than all other framings, aside from those framed in the context of ‘Conservation’ (median = 3.00, IQR = 2.50). Those framed solely around ‘Economics’ (median = 1.00, IQR = 0.25) and ‘Welfare’ (median = 1.00, IQR = 0.00) were significantly less common than those based on ‘Multiple frames’ or ‘Conservation’ alone.

**Figure 2.**
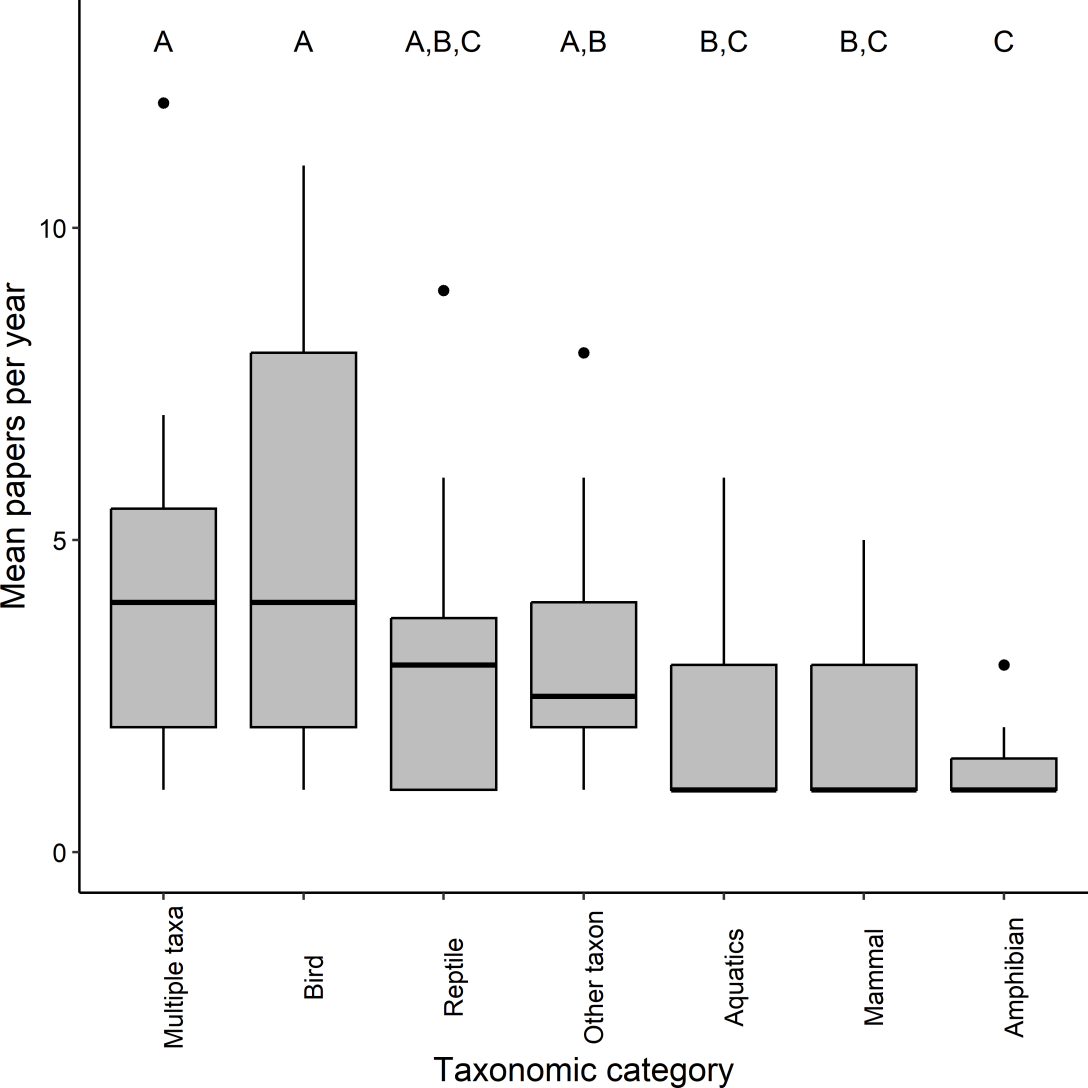
Mean peer-reviewed papers, per year, in each of the five taxonomic categories: Amphibians, Aquatics, Birds, Mammals and Reptiles—and Multiple taxa or Other taxon. Upper case letters indicate the grouping of framings based on significant differences found in a Kruskal–Wallis test.

Similarly, the number of peer-reviewed publications per year differed significantly among taxa according to the results of a Kruskal–Wallis test (*Χ*^2^ = 14.56, d.f. = 6, *p* = 0.024; figure 2). *Post hoc* tests show that peer-reviewed papers were more frequently focused on ‘Multiple taxa’ (median = 4, IQR = 3.5) or ‘Birds’ (median = 4, IQR = 6), than solely on ‘Aquatics’ (median = 1, IQR = 2), ‘Mammals’ (median = 1, IQR = 2) or ‘Amphibians’ (median = 1, IQR = 0.5). Those that focused on ‘Amphibians’ were also less common than peer-reviewed publications with no taxonomic focus (median = 2.5, IQR = 2). There were no other significant differences among categories.

### 6.2. Factors associated with citations per year

There was no correlation between the lead author h-index and journal impact factor with the latter being log transformed (*t* = 1.472, d.f. = 279, *p*‐value = 0.142, correlation coefficient = 0.088) or in its raw value (*t* = 1.134, d.f. = 279, *p*‐value = 0.190, correlation coefficient = 0.078) and so both variables were included in the analyses. The top performing model of citations per year contained the h-index of the first author, and the impact factor of the journal (AIC of 801.72; table 2). The next best performing model had a **∆**AIC of −4.46 relative to the best model, which suggests an equal level of support for both. This model additionally contained a term for the framing category suggesting an important role for this variable in determining the citation rate. The next best performing model was almost 9 AIC units higher (8.93), suggesting a large drop-off in model support.

**Table 2.** Linear mixed model outputs showing relative performance of models explaining the number of citations per year an article received (within peer-reviewed scientific literature with ‘year’ as a random effect). Bold text indicates models within 6 AIC units of the best model which were therefore equally supported.

model	d.f.	AIC	∆AIC	*R* ^2^
**first author h+impact factor**	**5**	**801.72**	**—**	**0.34**
**framing+first author h+impact factor**	**11**	**806.18**	**4.46**	**0.36**
framing+taxon + impact factor	16	815.11	13.39	0.36
taxon+first author h+impact factor	11	818.56	16.84	0.34
framing+taxon + first author h+impact factor+framing*taxon	41	821.15	19.43	0.37
framing+taxon + first author h+impact factor	17	822.94	21.22	0.37
framing+taxon + first author h+impact factor+impact factor*framing	23	831.00	29.28	0.37
framing+taxon + first author h+impact factor+framing*first author h	23	853.43	51.71	0.38
framing+taxon + first author h	16	876.83	74.81	0.18

**Figure 3.**
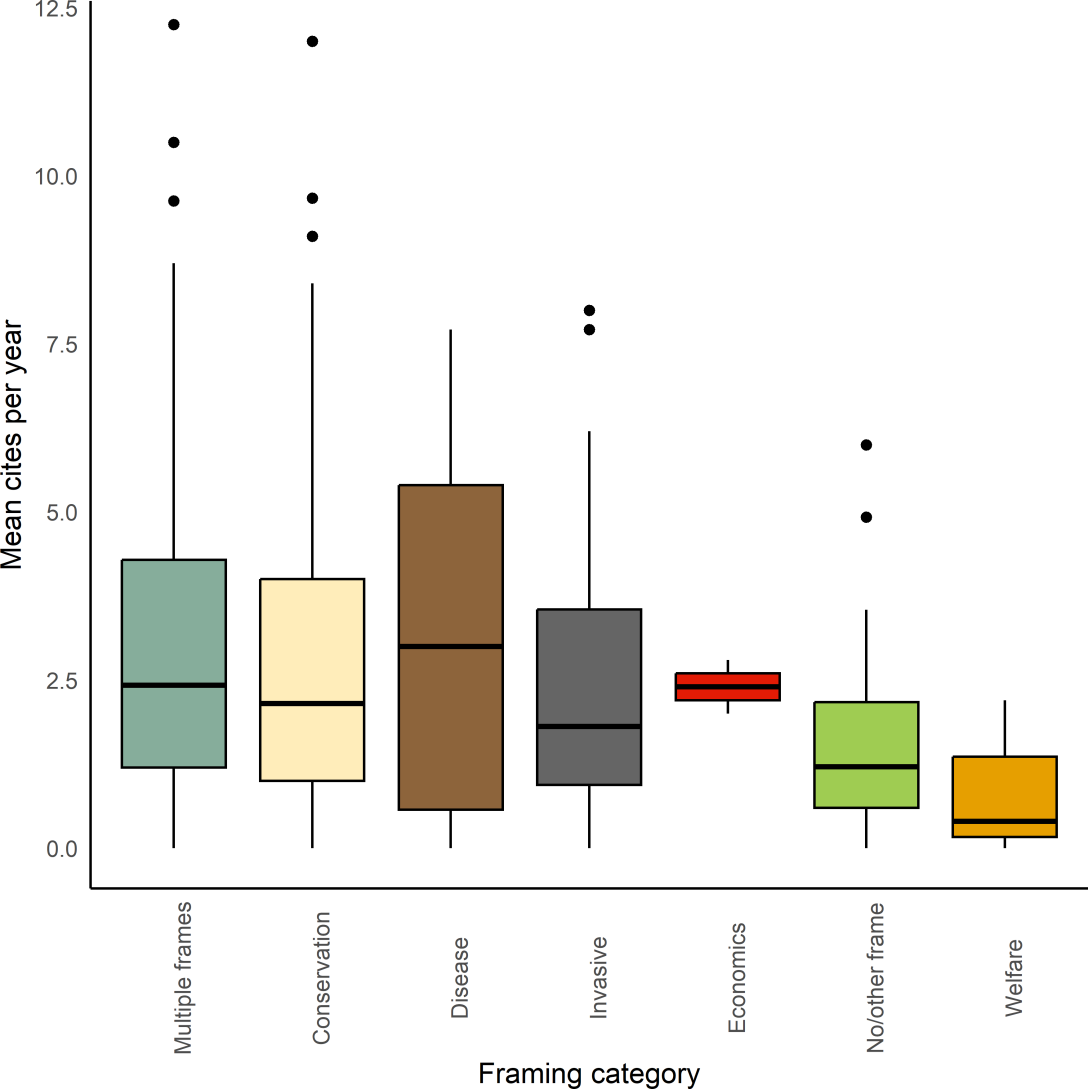
Mean citations for peer-reviewed papers, per year, for each of the five framing categories: Conservation, Disease, Invasives, Welfare and Economics—and those categories Multiple frames and No/other frame.

The ANOVA table of the model containing framing type, in addition the h-index of the lead author and journal impact factor, suggests a strong effect of each of these three variables on citation rate (framing category: *n* parameters = 6; *F* = 3.55; lead author: *n* parameters = 1; *F* = 8.43; impact factor: *n* parameters = 1; *F* = 77.79). Investigation of parameters from this model highlight a relatively strong effect size for the welfare framing compared with others, with an absolute *t*-value > 2.0, suggesting that peer-reviewed publications framed around welfare are cited fewer times per year than some other framings (table 3, figure 3). In particular, ‘Multiple frame’ peer-reviewed publications were cited more frequently that other categories, with a fourfold difference between them (mean = 3.44 citations per year) and the least cited framing (‘Welfare’, mean = 0.75 citations per year).

**Table 3.** Mean and s.d. values of peer-reviewed paper log transformed citations per year for each framing category, along with the parameter estimate for each from the best performing model. Framing categories are ordered in descending order of mean citations per year and those with *t*-values exceeding an absolute value of 2 are in bold.

	mean+/-s.d.	estimate	s.e*.*	*t-*value
Multiple framing	3.44 +/− 3.50	0.084	0.168	0.501
Conservation	3.40 +/− 3.89	−0.010	0.206	−0.050
Disease	3.05 +/− 2.50	0.100	0.268	0.365
Invasive	2.63 +/− 2.52	−0.217	0.271	−0.799
Economics	2.40 +/− 0.57	−0.013	0.739	−0.017
No clear framing	1.76 +/− 1.75	−0.394	0.295	−1.336
**Welfare**	**0.75 +/−0.81**	**−0.956**	**0.391**	**−2.443**
first author h-index		0.010	0.005	1.882
journal impact factor		0.759	0.088	8.590

**Figure 4.**
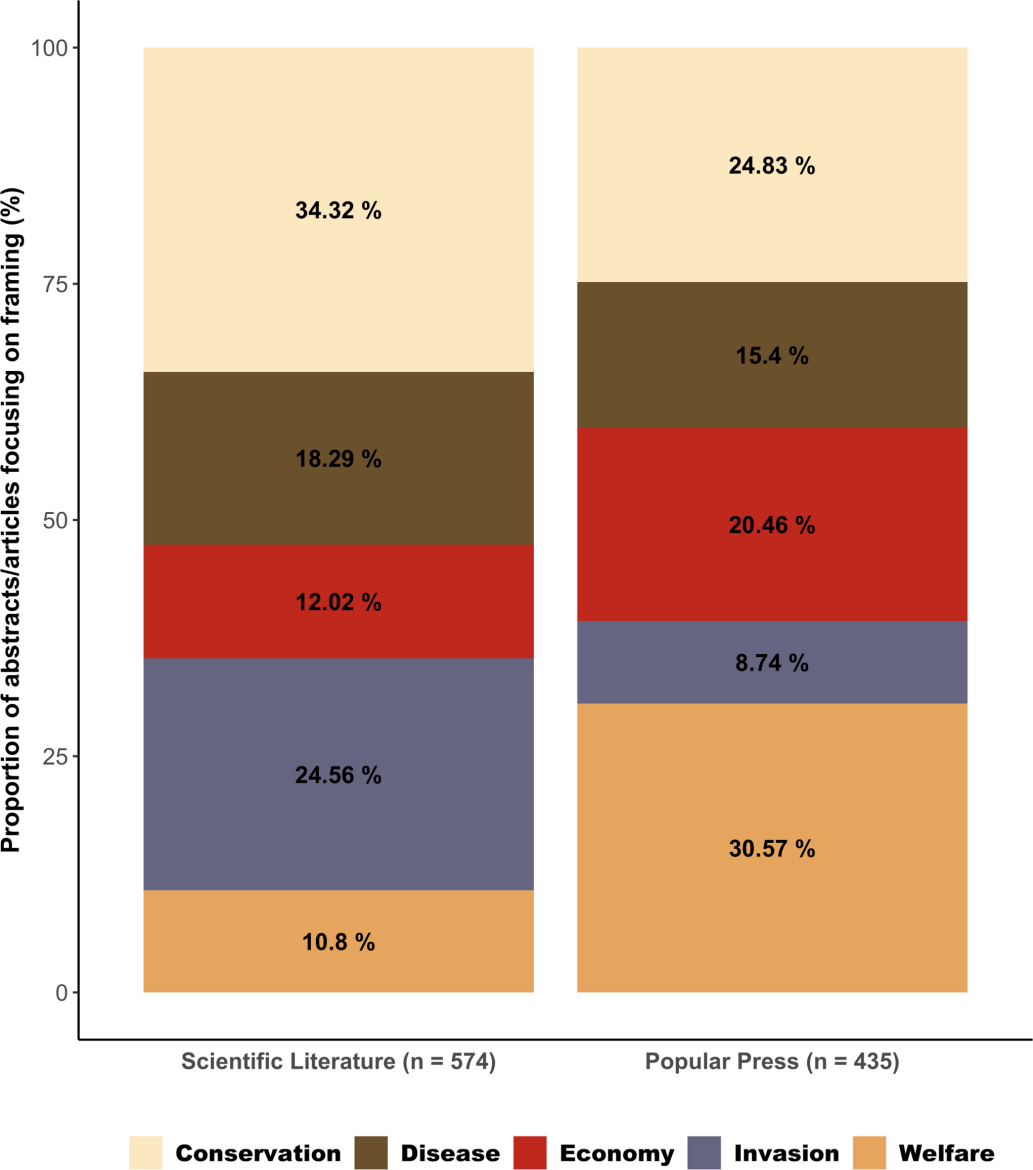
Proportion of each of the five framings in peer-reviewed scientific literature abstracts and newspaper articles. Abstracts/articles included in more than one framing are included in all for the purpose of this data visualization.

A total of 191 newspaper articles were included in our analyses (details of the articles in electronic supplementary material, S3 with the articles in S4). Of the five deductive themes used to categorize them ‘Welfare’ was the most common framing and had the most references to that framing within a given article (table 4). The frequency with which ‘Welfare’ and ‘Economy’ were used as a framing within newspaper articles compared with peer-reviewed publications were the most marked difference between the two media types (figure 4).

**Table 4.** Quantitative analysis of deductive and inductive coding from newspaper articles (*n* = 191). ‘Files’ refers to the number of articles in which the theme occurred, ‘references’ are the total number of items coded to that theme. A single news article may contain more than one of the subjects listed.

subject	files	references	mean refs
deductive			
Welfare	130	258	1.98
Conservation	106	166	1.57
Economy	89	153	1.72
Disease	65	139	2.14
Invasive	38	66	1.73

inductive			
Laws and Regulations	144	303	2.10
Public Health and Safety	126	298	2.37
Irresponsible Pet Ownership	77	137	1.78
Illegal Trade	66	101	1.53
Defence of Trade	31	48	1.55

‘Conservation’ was proportionally the highest framing in peer-reviewed publications (34.3% of abstracts) and second highest in newspaper articles (24.8% of articles), but still exhibited a percentage point reduction of almost 10% between the two media types. In contrast, ‘Welfare’ was the least frequently used frame in peer-reviewed publications and highest in newspapers (10.8% compared with 30.6%) and represents the largest change of any frame between the two media types. When inductive categories were also included, the most frequent framings in popular media were ‘Laws and Regulations’ and ‘Public Health and Safety’, both of which were used as framings at a comparable frequency to ‘Welfare’ in the deductive categories. For newspaper articles within these framings the topics were also referenced frequently (table 4). For each category all thematically mapped text is available in the supplementary information of this article [45] and [46].

### 6.3. Comparison of language between the two media types

**Figure 5.**
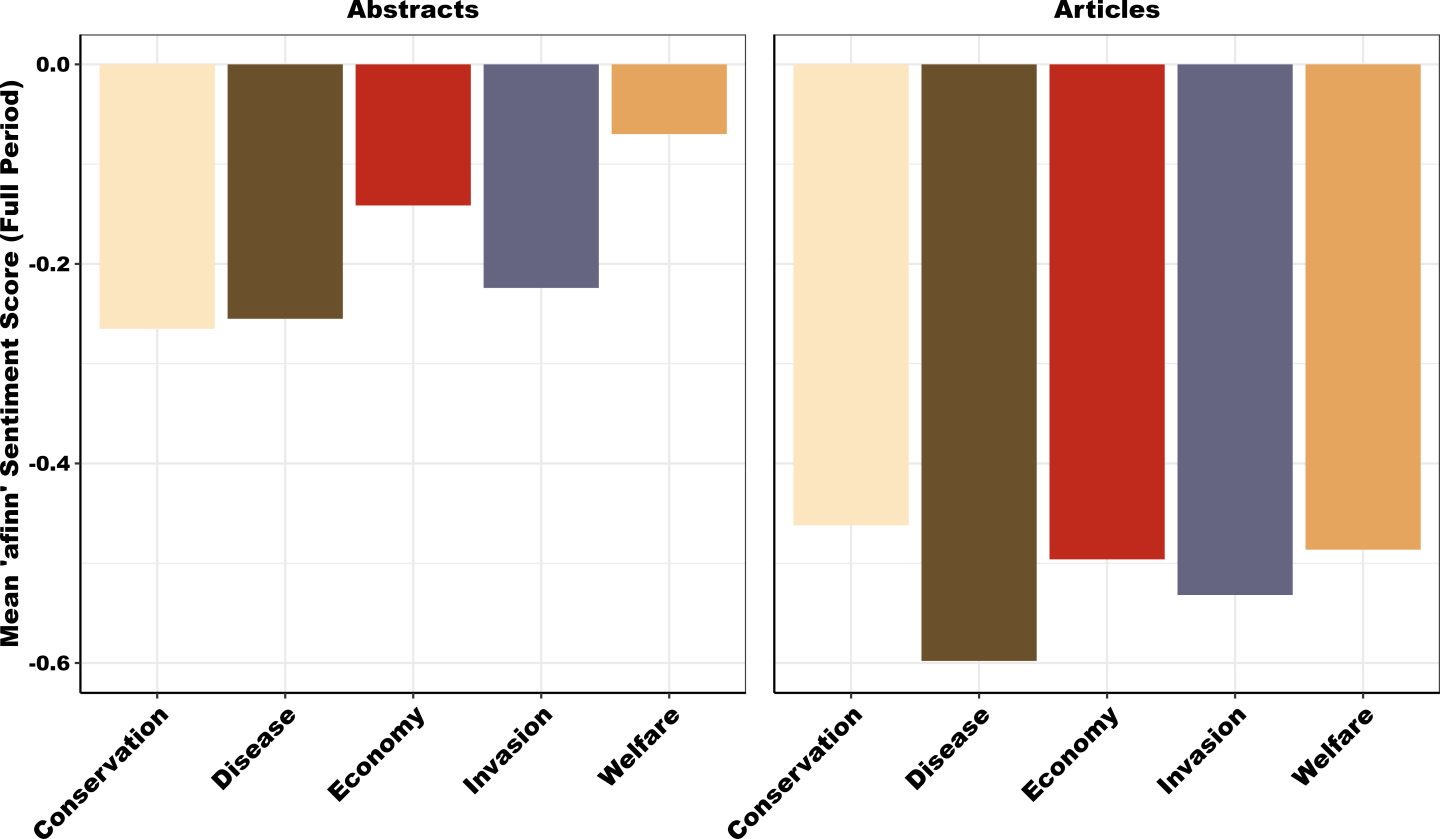
Mean AFINN sentiment scores among the five framing categories for peer-reviewed abstracts (left) and newspaper articles (right). Peer-reviewed abstracts typically contained less negative language from the AFINN lexicography than newspaper articles. There was relatively more variation in sentiment among framings in peer-reviewed abstracts, with Economics and Welfare being less negative than other framings.

AFINN sentiment analyses (figure 5) suggests that the language used in newspapers was substantially more negative than that used in the abstracts of peer-reviewed papers. Within a single media type, there were also notable differences in language use between framing categories. For example, within abstracts framed around ‘Welfare’, there was less negative language than other categories, particularly those framed around ‘Conservation’, ‘Disease’ and ‘Invasive species’. In newspapers articles the differences in sentiment between categories was relatively smaller.

NRC sentiment scores suggest some consistency in emotive language category use for a given framing, and among framing types within a media type. However, at the broadest scale between media types there is a great deal of variation in language category used. For example, within newspaper articles the following language types are greater than that seen in abstracts: ‘Anger’, ‘Sadness’, ‘Fear’, ‘Joy’ and ‘Anticipation’ (table 5). Peer-reviewed abstracts are higher for ‘Trust’ and ‘Positive’ language. There is no variation between the two media types for ‘Negative’ language, ‘Disgust’ or ‘Surprise’. Examples of a selection of articles scoring highly for these emotion categories are available in electronic supplementary material, table S6, and example of newspaper article and peer-reviewed abstract on a similar subject are available in the electronic supplementary material, S1 section ‘Example of narrative-driven newspaper article versus data-driven peer-reviewed abstract’.

**Table 5.** Comparison of percentage of newspaper articles and peer-review publication abstracts consisting of words in each of the NRC language type categories. We have defined there to be a difference between the two media types when the range of percentages do not overlap between the media types.

	news articles (%)	scientific abstracts (%)	variation?
Anger	6.2−6.5	4.7−5.3	newspaper articles higher %
Anticipation	8.2−8.5	7.0−8.1	newspaper articles higher %
Fear	11.4−12.1	8.1−11.0	newspaper articles higher %
Joy	5.2−5.8	3.9−4.7	newspaper articles higher %
Sadness	7.1−7.6	5.0−6.9	newspaper articles higher %
Positive	20.4−21.2	24.2−27.6	peer-review abstracts higher %
Trust	12.0−12.6	14.5−17.5	peer-review abstracts higher %
Disgust	4.7−5.7	3.7−5.0	overlaps – no difference
Negative	18.2−19.0	16.9−18.6	overlaps – no difference
Surprise	3.7−4.0	3.8−4.1	overlaps – no difference

**Figure 6.**
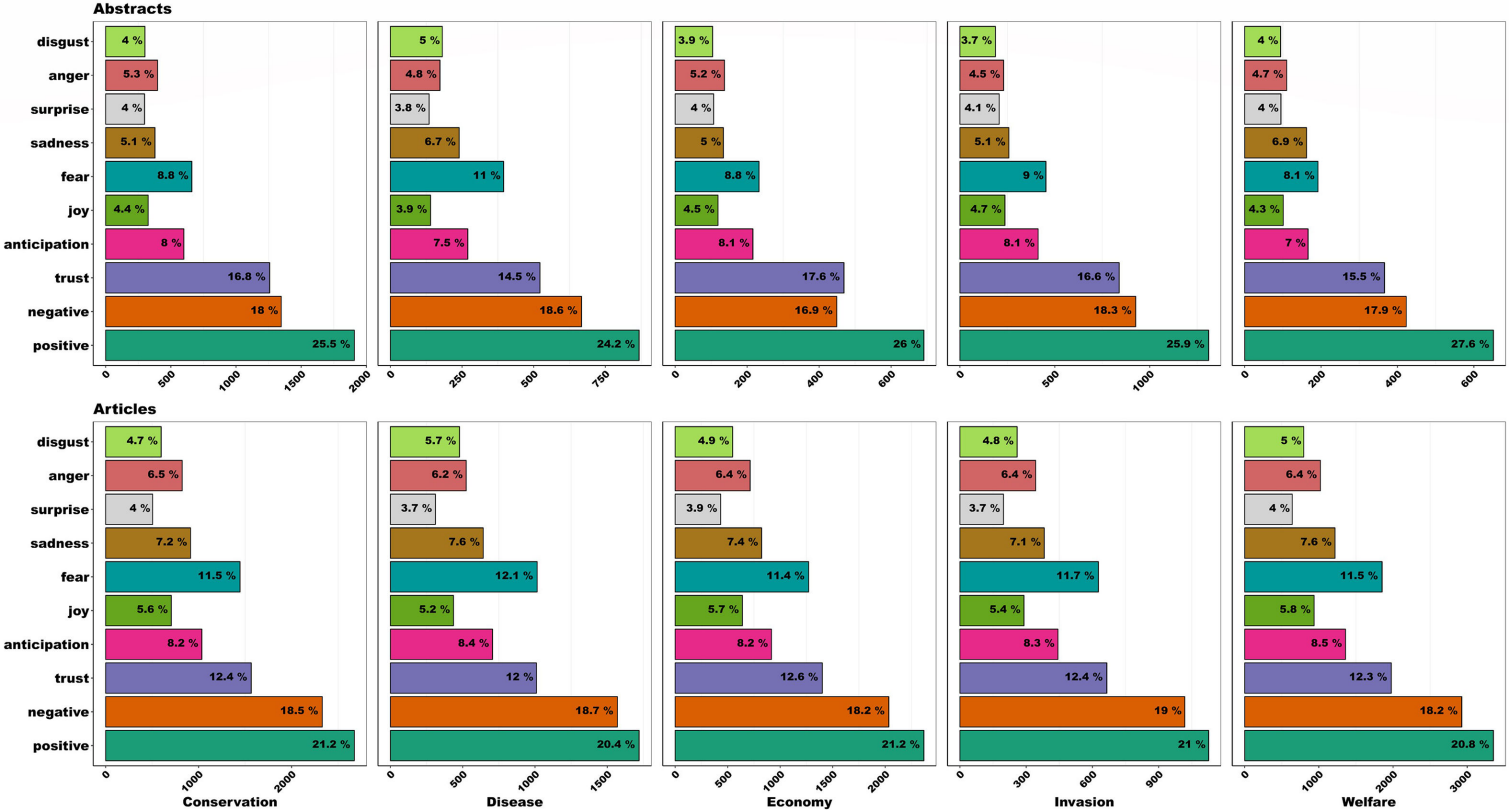
NRC sentiment scores (%) for all five framings for both peer-reviewed abstracts (top) and newspaper articles (bottom). Profiles and relative ranks of each of the eight emotions and two sentiments (positive/negative) encoded in the NRC lexicography are consistent across all combinations of framing and media type. Note the *x*-axis changes scale as the absolute number of words varies between framings.

The shape of the profiles of relative use of the categories are largely consistent, meaning that the relative order of emotional language used (but not absolute levels—see table 5) was comparable between framings categories and media types (figure 6). In all 10 combinations of framing and media type, the five most used categories of words were consistent. In all 10 combinations of framing and media type, the three most used language categories were used in over 50% of the words in those articles.

## 7. Discussion

The number of peer-reviewed publications focusing on the exotic pet trade has increased greatly since the turn of the century. Our analyses suggest that certain taxonomic and framing biases exist in their frequency of use and citation rate within peer-reviewed papers, and that these biases in framing extend and contrast with those observed in newspapers. There were also differences in language used between framings and media types, with newspaper articles being written in more negative, emotive language. The potential drivers of these patterns and their implications are discussed below.

Our findings in peer-reviewed publications highlight that welfare was under-represented, consistent with previous research [30], suggesting that relatively little change has occurred in this area in the past decade. We propose two possible underlying reasons for this: our poor knowledge of trade dynamics, and the fundamental difficulties of measuring the welfare of an animal. In the case of the former, significant knowledge gaps exist in our understanding of trade supple chain structures and operations [47–49], and how drivers of trade may result in different dynamics even for closely related species [50]. These difficulties are amplified because of the illicit nature of a large proportion of wildlife trade, which makes it inherently hard to monitor and quantify [51]. Additionally, accurately measuring welfare in some taxa is hard. Reptile species, for example, are traded in extremely large numbers [52], but our results highlight that scientific literature on their welfare is lacking. While our knowledge in this area is improving (e.g. [53]), we still lack holistic metrics of welfare for many taxa and species that are traded in great numbers as pets, and perhaps do not implement the simpler physical metrics of health, or even mortality rates in transit, as widely or effectively as we could [54]. In the absence of a way to improve our knowledge of welfare in the exotic pet trade and how to accurately measure it, one option would be to apply the precautionary principle and aim to improve welfare wherever possible, even in the absence of data suggesting it to be an immediate issue.

In contrast to peer-reviewed articles, newspaper articles were most frequently framed in the context of animal welfare. This may be due to the structure and nature of the media in question. The more story-like structure of newspaper articles is more amenable than a more neutral peer-reviewed paper to a narrative welfare piece focused on the experience of an individual pet or its guardian. Additionally, given the nature of the two types of media it was no surprise that their language use differed, with more negative, emotive language being more likely in newspapers articles. The individual-level focus of newspaper articles may also explain why this platform commonly used frames that were not found in the titles or abstracts of peer-reviewed papers: ‘Laws and Regulations’ and ‘Public Health and Safety’. For example, emphasis on information related to the legality of the pet trade and the risk it poses to public health has been linked to a reduction in the likelihood of consumers purchasing exotic pets [24], highlighting the individual-level influence these framings can have, and hence their popularity as a framing tool in newspaper articles.

The disjunct between welfare coverage in peer-reviewed papers and newspaper articles, and the language use within them, could have considerable implications. The coverage and framing of a subject within the popular media can drive a demand for specific, high visibility ‘solutions’ to an issue [23] that, while understandable and well-intentioned, may in reality be damaging to efforts and interventions aimed to develop sustainable and more ethical trade [55,56]. For example, several articles in our newspaper dataset advocated a ban to end the exotic pet trade, and wildlife trade more generally, despite evidence that such bans may have unintended negative side-effects [57]. This example highlights the need to take a more integrated approach to tackling the challenges posed by the exotic pet trade. Such an approach will require a synthesis of peer-reviewed evidence, public engagement and learning from other disciplines seeking to change the behaviours of both consumers and suppliers [58].

Conservation was a frequently used frame in both media types, probably due to the current biodiversity crisis and its societal threat. Conservation tends to focus at the level of the species or population [59] and may be perceived to be a larger issue in scale and importance. There have been many calls to find common ground and goals between the two disciplines [60,61] that have until recently been considered quite disparate [62,63]. As Baker *et al*. highlight, this tendency not only exists in the literature, but also in trade monitoring, as exemplified in Convention on International Trade in Endangered Species (CITES) articles and their interpretation and reporting [30,64]. Very few analyses of CITES and its role in the wildlife trade refer to the welfare of the animals involved despite the existence of welfare controls within the relevant articles (e.g. Article IV on the control of Appendix II species: ‘The export of any specimen of a species included in Appendix II shall require the prior grant and presentation of an export permit. An export permit shall only be granted when the following conditions have been met: … a Management Authority of the State of export is satisfied that any living specimen will be so prepared and shipped as to minimize the risk of injury, damage to health or cruel treatment’ [65]). Better coordination between the two disciplines would be beneficial, perhaps starting with more consistent communication of already-existing welfare considerations within CITES, but extending to more specific welfare considerations and legislation where it is lacking in the legal trade.

There were other framing biases and gaps in peer-reviewed literature. The sparse use of the framing around economics of the exotic pet trade was notable given its size and the evidence for benefits of sustainable use in some parts of the pet trade [9] as well as the more general use of wildlife [55]. Under sustainable-use models, components of biodiversity are used at a rate that does not lead to its long-term decline. It therefore maintains its potential to meet the needs of present and future generations [66]. The strategy can be particularly important for supporting local livelihoods and engendering equity of roles, rights and agency of peoples within range states [67]. However, there are relatively few empirical examples linking the pet trade to the benefit of member state communities or written within the context of longer-term sustainability of traded populations [68,69]. Where such studies do exist, they often mention the limited scope of the pet trade in poverty alleviation, or an associated lack of motivation for effective stewardship of traded species [8,70]. In the wake of the COVID−19 pandemic the potential role of the wildlife trade in pathogen spillover has received increased levels of attention [55,71]. Although public health and safety was a common framing in newspaper articles, the equivalent framing in peer-reviewed papers (disease) was not so common. Were these analyses revisited to include peer-reviewed papers post−2020 it seems highly likely that the perceived public health risks (and pathogen spillover risk more generally) of the pet trade would be more highly emphasized.

In terms of taxonomic trends, reptiles and birds (particularly parrots) are most likely to be threatened by extinction because of overuse [16], with many species and individuals being traded as pets [2,72]. However, the prevalence of these taxa within the peer-reviewed literature does not directly correspond with their volumes in the trade. Birds are commonly the focus of conservation-framed papers; reptiles are much less so. These biases are probably a result of interacting factors, including our baseline level of knowledge of and interest in birds compared with other groups [66], which in part is fed by societal preferences towards certain taxa [73]. A key knowledge gap reflected in our own approach is the bias towards vertebrates in studies (including this one), which reflects broader taxonomic and geographic inequalities in wildlife trade data and attention [74], and the existence of which has led to calls for a more inclusive approach to trade management [75]. Certain invertebrates [16,76], plants [72,77] and fungi [78] are heavily impacted by collection for the pet or horticulture trade, and as outlined by Hinsley *et al*. [75] ‘these biases can hamper effective policy interventions, reduce awareness of wider threats from trade, and prevent conservation efforts from focusing on the most pressing issues’.

There are a number of ways in which the findings of this research could be further developed to provide a broader and deeper look at how communication style affects the engagement with content on wildlife trade, and whether this engagement translates to a positive impact on behaviour change. We were unable to quantify the level of engagement with newspaper articles, but use of a suitable metric would give a better understanding of the impact of framings and language use in comparison with those of peer-reviewed papers. Further, by including additional popular media channels, such as television, social media, blogs, online forums and advocacy documents, a more holistic view of the patterns and trends in communication, as well as their impact could be obtained. Within the parameters of our study, our choice of focusing solely on the title, keywords and abstract of scientific papers could have polarized our results: the abstract is typically the densest, driest part of a peer-reviewed paper, and further research looking at text within the discussion could be an interesting avenue for future research. Similarly, our choice of search terms, although iteratively developed to maximize their efficacy will inevitably lead to some biases in our findings. In terms of obtaining a more global view of the issue of communication in this subject area, a great deal of the impact of the pet trade occurs at the national and local level [16] in countries in which English is not the first language. Given the importance of national and local trade it seems likely that similar, finer-scale, native language studies may yield interesting and useful results. Such approaches could examine cultural similarities [10,11] and differences in attitudes towards keeping exotic pets [79], the acceptance and use of fundamental concepts related to the trade [80], and how these may change with stakeholder demographics [81,82]. Any such cultural and demographic differences must be acknowledged and understood if in-country and local trade is to be developed in a sustainable and beneficial way [16].

Overall, this study presents an effort to monitor patterns of communication and focus on the global pet trade in two media types, but to enhance its impact more specific research and interventions will be needed. Identifying communication patterns on different forms of media (social media posts, forums, blogs), will provide a more holistic view of the subject. In turn, it is important to understand and discuss how these patterns can support or counter efforts to introduce sustainable behaviour change. There also remain significant biases and knowledge gaps that require addressing in a strategic way in order to better focus efforts of conservation and welfare practitioners on the most pressing and severe issues.

## Data Availability

All supplementary information associated with this article, including the datasets are available in Bielby et al. 2025 [45,46].
